# Supplementary material for: The autonomy paradox in AI-generated content adoption: Creative-specific alternative to TAM model in China’s micro-short drama industry
Source: PLoS One. 2026 Jan 30;21(1):e0336166. doi: 10.1371/journal.pone.0336166 (PMC12858070; doi:10.1371/journal.pone.0336166)
Supplement: S1 File — Contains the complete bilingual (Chinese-English) questionnaire used in the quantitative study, including all items for Innovation Compatibility (IC), Creative Autonomy Retention (CAR), Adoption Intention (AI), and Actual Usage Behavior (AUB), along with demographic questions. (DOCX) [file pone.0336166.s001.docx]

### **Questionnaire Instrument**

**Title:** Survey on the Adoption of AIGC in Micro-Short Drama Creation

Introduction & Consent

Dear Micro-Short Drama Creator:

You are invited to participate in an academic study regarding the adoption of Artificial Intelligence Generated Content (AIGC) technology in the creation of micro-short dramas. This survey aims to understand creators’ acceptance of AIGC tools and their impact on creative autonomy.

The survey will take approximately **5–10 minutes** to complete. Your participation is strictly voluntary. All data collected will remain **anonymous and confidential**, used exclusively for academic research purposes.

By proceeding with this survey, you indicate that you have read the above information and voluntarily agree to participate.

**Part I: Basic Information**

1. Gender:

□ Male □ Female □ Other / Prefer not to say

2. Age:

□ Under 25 □ 25–34 □ 35–44 □ 45 and above

3. Creative Experience:

□ Less than 1 year □ 1–3 years □ 4–6 years □ More than 7 years

4. Primary Role:

□ Screenwriter □ Director □ Editor □ Independent Creator □ Other

5. Commonly Used AIGC Domains:

□ Script Generation □ Visual Design □ Post-production Effects □ Audio Production □ Other

**Part II: Core Measurement Scales**

Please rate the following statements based on your actual experience:

(1 = Strongly Disagree, 2 = Disagree, 3 = Neutral, 4 = Agree, 5 = Strongly Agree)

Innovation Characteristics (IC)

6. IC1: AIGC has brought revolutionary innovation to micro-short drama creation.

7. IC2: AIGC offers unique advantages that traditional creative tools cannot replace.

8. IC3: The rapid advancement of AIGC technology provides more possibilities for creation.

9. IC4: The creative values of AIGC align highly with my own creative pursuits.

Creative Autonomy & Agency (CAR)

10. CAR1: When using AIGC, I retain full control over core creative decisions.

11. CAR2: AIGC enhances rather than replaces my ability to express creativity.

12. CAR3: I can effectively balance AIGC assistance with my own originality.

13. CAR4: Sometimes, AIGC limits my creative autonomy, preventing me from having full control over decisions. (Reverse-coded)

14. CAR5: I have sufficient control and freedom to edit or modify AIGC-generated content.

Adoption Intention (AI)

15. AI1: I plan to continue using AIGC in future micro-short drama projects.

16. AI2: I am willing to recommend AIGC tools to my peers.

17. AI3: I intend to devote more time to learning advanced AIGC skills.

18. AI4: I believe AIGC will become an essential part of my creative process.

Actual Usage Behavior (AUB)

19. AUB1: I use AIGC tools in the majority of my micro-short drama projects.

20. AUB2: AIGC has become an indispensable tool in my creative workflow.

21. AUB3: I actively explore new AIGC functions and application scenarios.

**Part III: Open Feedback**

Please briefly share a specific case of your AIGC usage and how it influenced your creative autonomy (Optional, 50–100 words):

**— End of Survey. Thank you for your participation! —**
