# Supplementary material for: The autonomy paradox in AI-generated content adoption: Creative-specific alternative to TAM model in China’s micro-short drama industry
Source: PLoS One. 2026 Jan 30;21(1):e0336166. doi: 10.1371/journal.pone.0336166 (PMC12858070; doi:10.1371/journal.pone.0336166)
Supplement: S2 File — Comprehensive documentation of the scale development process for all CITAM constructs, with detailed focus on the Creative Autonomy Retention (CAR) scale. Includes theoretical grounding, item generation, pilot testing, expert reviews, and psychometric validation. (DOCX) [file pone.0336166.s002.docx]

# Creative Industry Technology Acceptance Model (CITAM) Scale Development

## B.1 Introduction and Theoretical Foundation

The Creative Industry Technology Acceptance Model (CITAM) integrates four core constructs to explain the adoption mechanisms of AIGC in the creative industry. Creative Autonomy Retention (CAR) is an original construct of this study, conceptualized as creators' perceived ability to maintain control over the creative process, sense of agency, and psychological ownership when integrating AIGC tools. This construct is primarily based on Self-Determination Theory (SDT; Deci & Ryan, 2000; Ryan & Deci, 2020), which emphasizes autonomy as a fundamental psychological need for intrinsic motivation. Innovation Compatibility (IC) is based on Rogers' (2003) Diffusion of Innovations Theory, measuring the degree of fit between AIGC tools and creative workflows. Adoption Intention (AI) and Actual Use Behavior (AUB) are adapted from Davis's (1989) TAM model, measuring creators' behavioral intentions and actual usage behaviors respectively.

CITAM represents an alternative to TAM specifically tailored for creative contexts, focusing on autonomy retention in AI-assisted environments rather than traditional usefulness and ease-of-use perceptions. The model integrates elements from psychological ownership theory (Pierce et al., 2001) and creative identity theory (Petkus, 1996). It addresses the limitations of traditional technology acceptance models by capturing the "autonomy paradox" where AIGC improves efficiency but may threaten creators' sense of control - a dynamic not adequately captured by TAM's original constructs.

Scale development follows DeVellis's (2016) eight-step scale construction process, ensuring theoretical foundation, content validity, and psychometric robustness. This approach aligns with SSCI standards for measuring new constructs in adoption research (e.g., Venkatesh et al., 2016).

## B.2 Step-by-Step Development Process

### 1. Determining Measurement Content (Conceptual Definition)

Based on literature review, the four constructs of CITAM are defined as follows:

**CAR (Creative Autonomy Retention)**: A unidimensional construct encompassing three aspects: (a) control over core decisions (such as final creative output), (b) psychological ownership (such as the sense of "my creation"), and (c) agency in AI-human collaboration (such as AI as tool rather than replacement). Key references include SDT's autonomy need (Ryan & Deci, 2020), Job Demands-Resources Model (Bakker & Demerouti, 2017), and AI-specific human-machine teaming research (Seeber et al., 2020).

**IC (Innovation Compatibility)**: Based on Rogers' (2003) Diffusion of Innovations Theory, measuring the degree of consistency between AIGC and existing creative practices, values, and workflows.

**AI (Adoption Intention)**: Based on TAM and UTAUT theories, measuring creators' behavioral intentions to adopt and continue using AIGC tools.

**AUB (Actual Use Behavior)**: Measuring creators' actual usage frequency and extent of AIGC tools in daily creative practices.

### 2. Generating Item Pool

Initial item pools were generated for the four constructs through the following methods:

**CAR Construct**: Initial 12 items generated through (a) adapting existing scales (such as SDT's perceived autonomy scale), (b) expert brainstorming (3 creativity researchers and 2 MSD practitioners), and (c) focus group discussions (n=10 creators via WeChat).

**IC Construct**: Initial 8 items adapted from Rogers (2003) and Moore & Benbasat (1991) compatibility scales, combined with AIGC and micro-short drama creation contexts.

**AI Construct**: Initial 6 items adapted from Davis (1989) TAM and Venkatesh et al. (2003) UTAUT behavioral intention scales.

**AUB Construct**: Initial 5 items developed based on TAM's actual use behavior and related technology use behavior research.

All items were expressed using simple, context-specific language, employing a 5-point Likert scale (1=strongly disagree to 5=strongly agree).

### 3. Determining Measurement Format

Reflective measurement models were selected for all constructs, where items reflect the latent constructs (Jarvis et al., 2003). Reverse-coded items were included in the CAR scale to mitigate acquiescence bias. Bilingual (Chinese-English) versions were prepared for all scales with back-translation to ensure equivalence.

### 4. Content Validity Expert Review

Five experts (2 psychology experts, 2 information systems experts, 1 creative industry expert) rated the relevance and clarity of items for all four constructs using Content Validity Ratio (CVR; Lawshe, 1975). Items with low CVR were deleted or revised:

- CAR: Reduced from 12 to 8 items
- IC: Reduced from 8 to 6 items
- AI: Reduced from 6 to 5 items
- AUB: Maintained 5 items

### 5. Pilot Study and Exploratory Factor Analysis (EFA)

A pilot study was conducted with 50 MSD creators. EFA results confirmed the factor structure of each construct:

**CAR**: Unidimensionality confirmed (KMO=0.82; Bartlett's test p<0.001; single factor eigenvalue=3.45, explaining 68% variance), 3 low-loading items deleted, 5 items retained, Cronbach's α=0.87.

**IC**: Unidimensionality confirmed, 2 low-loading items deleted, 4 items retained, Cronbach's α=0.85.

**AI**: Unidimensionality confirmed, 1 low-loading item deleted, 4 items retained, Cronbach's α=0.82.

**AUB**: Unidimensionality confirmed, 2 low-loading items deleted, 3 items retained, Cronbach's α=0.81.

### 6. Main Study Validation (Confirmatory Factor Analysis, CFA)

In the main sample (n=607), overall measurement model CFA conducted through AMOS confirmed excellent model fit: χ²/df=1.834, CFI=0.988, TLI=0.985, RMSEA=0.037, SRMR=0.030.

**Convergent Validity for Each Construct**:

- CAR: AVE=0.702, CR=0.922, all loadings >0.80
- IC: AVE=0.713, CR=0.908, all loadings >0.82
- AI: AVE=0.696, CR=0.890, all loadings >0.82
- AUB: AVE=0.696, CR=0.872, all loadings >0.83

**Discriminant Validity**: All constructs satisfied the Fornell-Larcker criterion, with HTMT ratios all below the 0.85 threshold.

**Nomological Validity**: Inter-construct correlations aligned with theoretical expectations, supporting hypothesized relationships.

### 7. Reliability and Validity Assessment in Context

- **Test-Retest Reliability**: Established through subsample (n=30) at two-week intervals, all constructs r>0.80.
- **Criterion Validity**: Each construct significantly correlated with related outcome variables, supporting predictive validity.
- **Common Method Bias**: Not a major issue based on Harman's single-factor test (first factor explained variance <50%).

### 8. Final Scale Refinement

The final CITAM four-construct scale was retained with 16 items total (CAR: 5 items, IC: 4 items, AI: 4 items, AUB: 3 items). The scale is concise, reliable, and valid for measuring AIGC adoption mechanisms in the creative industry.

## B.3 Final Items

### Creative Autonomy Retention Scale (CAR) - 5 Items

**Cronbach's α = 0.922, CR = 0.922, AVE = 0.702**

| **Item Code** | **English Version** | **Theoretical Dimension** | **Factor Loading** |
| --- | --- | --- | --- |
| CAR1 | When using AIGC, I can still fully control the core creative decisions. | Control | 0.804 |
| CAR2 | AIGC enhances rather than replaces my creative expression ability. | Agency | 0.817 |
| CAR3 | I can effectively balance the relationship between AIGC assistance and originality. | Agency/Control | 0.856 |
| CAR4® | Using AIGC sometimes limits my creative autonomy, preventing me from fully controlling decisions. (Reverse-coded) | Control | 0.853 |
| CAR5 | I have full editorial and modification control over the content generated by AIGC. | Control/Ownership | 0.858 |

### Innovation Compatibility Scale (IC) - 4 Items

**Cronbach's α = 0.901, CR = 0.908, AVE = 0.713**

| **Item Code** | **English Version** | **Factor Loading** |
| --- | --- | --- |
| IC1 | AIGC brings revolutionary methods of innovation to micro-short drama creation. | 0.823 |
| IC2 | AIGC tools are highly compatible with my existing creative workflows. | 0.856 |
| IC3 | Using AIGC aligns with my needs and expectations for efficient creation. | 0.871 |
| IC4 | AIGC is consistent with my creative values and working methods. | 0.827 |

### Adoption Intention Scale (AI) - 4 Items

**Cronbach's α = 0.887, CR = 0.890, AVE = 0.696**

| **Item Code** | **English Version** | **Factor Loading** |
| --- | --- | --- |
| AI1 | I intend to continue using AIGC in my future micro-short drama projects. | 0.821 |
| AI2 | I would recommend AIGC tools to my colleagues for creative work. | 0.847 |
| AI3 | I plan to increase the frequency of AIGC use in my creative process. | 0.834 |
| AI4 | I have a strong motivation to adopt AIGC technology in my creative work. | 0.835 |

### Actual Use Behavior Scale (AUB) - 3 Items

**Cronbach's α = 0.870, CR = 0.872, AVE = 0.696**

| **Item Code** | **English Version** | **Factor Loading** |
| --- | --- | --- |
| AUB1 | I use AIGC tools in most of my micro-short drama projects. | 0.835 |
| AUB2 | I frequently use AIGC to assist my creative work. | 0.842 |
| AUB3 | AIGC has become an indispensable part of my daily creative workflow. | 0.827 |

## B.4 Limitations and Future Improvements

The CITAM scale is specifically designed for AIGC applications in creative industries; its generalizability to non-creative domains requires further validation. While each construct demonstrates strong unidimensional psychometric properties, future research could explore potential multidimensional structures. Additionally, testing the scale in different cultural contexts outside China would further enhance its robustness and external validity.

**Notes**:

- All items use a 5-point Likert scale (1=strongly disagree to 5=strongly agree)
- ® indicates reverse-coded item (CAR4)
- CFA loadings are standardized estimates from the main study (n=607)
- Overall model fit: χ²/df=1.834, CFI=0.988, TLI=0.985, RMSEA=0.037, SRMR=0.030
