# Supplementary material for: The autonomy paradox in AI-generated content adoption: Creative-specific alternative to TAM model in China’s micro-short drama industry
Source: PLoS One. 2026 Jan 30;21(1):e0336166. doi: 10.1371/journal.pone.0336166 (PMC12858070; doi:10.1371/journal.pone.0336166)
Supplement: S3 File — Contains the interview outline (question guide) and complete de-identified transcripts from 10 creative professionals. All potentially identifying information has been removed to protect participant privacy. (DOCX) [file pone.0336166.s003.docx]

# Interview Guide

**Introduction:**
Hello! Thank you very much for participating in this interview. We are conducting a study on the application of AIGC technology in video content creation, aiming to understand how pioneering companies like Xuanjia Technology collaborate with AIGC tools. Your valuable experience and insights are critical to our research. This interview will remain anonymous, and all information will be used solely for academic purposes. Please feel free to express your thoughts openly.

### Part I: Personal Background and Overview of AIGC Use

**Q1:** Could you briefly introduce your position, main responsibilities, and years of work experience at Xuanjia Technology?
**Q2:** In your daily work, which AIGC tools or platforms do you mainly use? (e.g., Kino platform, Juhaojian, other AIGC tools)
**Q3:** How often and for how long do you typically use AIGC tools? (e.g., daily, several times per week, only for specific projects)

### Part II: Perceived Usefulness and Ease of Use (PU & PEOU)

**Perceived Usefulness (PU):**
**Q4:** In your opinion, in what ways does AIGC (especially the Kino platform) help you most in your work? Which pain points does it address? (e.g., material generation, scriptwriting, video editing, efficiency, cost reduction)
**Q5:** How do you think AIGC tools perform in improving work efficiency, enhancing output quality, or helping you achieve new creative goals? Could you share a specific example?
**Q6:** Which functions of AIGC tools do you find “less useful” or “not effective”? Why?
**Q7:** From a technical perspective, in what areas does the Kino large model provide unique value for video content creation? How does it compare to other general-purpose large models?

**Perceived Ease of Use (PEOU):**
**Q8:** Do you find the user interfaces of AIGC tools friendly? How is the learning curve?
**Q9:** What difficulties have you encountered while using AIGC tools? (e.g., technical issues, complex operations, lack of learning resources) How did you overcome them?
**Q10:** How would you evaluate your company’s training and technical support for AIGC tool usage? What improvements do you suggest?

### Part III: Perceived Creative Enhancement (CEP)

**Q11:** How have AIGC tools (e.g., Kino’s ability to generate videos from images and text outlines) influenced your creative process? Have they inspired your imagination or sometimes limited it? How do they help you achieve breakthroughs in visual presentation, composition, or style exploration?
**Q12:** Has AIGC provided you with new perspectives or solutions in script development, plot progression, or character dialogue?
**Q13:** How do you evaluate AIGC tools in terms of content originality and diversity? Can you create works with personal style or uniqueness using AIGC?
**Q14:** Do you think AIGC has enabled you to devote more energy to valuable creative ideation and conceptual design while reducing repetitive labor? Could you share an example?
**Q15:** To what extent do you think AIGC enhances the overall creative quality of your work? Does it help you achieve higher artistic standards?

### Part IV: Perceived Creative Autonomy (CAR)

**Q16:** In collaborating with AIGC, do you feel your sense of “control” over the work has changed? Does this change make you feel freer or more restricted?
**Q17:** Are you concerned that the “black-box nature” of AIGC may affect your control over the final work?
**Q18:** How do you think the widespread use of AIGC will impact your role (e.g., 3D designer, editor) in the future? Do you worry about your professional skills being replaced or devalued?
**Q19:** How do you view the division of roles and collaboration between AI and human creators? To what extent do you think AI will gain “autonomy” in the creative process in the future?
**Q20:** When AIGC-generated content does not match your expectations, how do you handle it? Do you feel you are merely “adjusting” AI’s output rather than “creating” from scratch?
**Q21:** What are your views on the authorship or attribution of AIGC-assisted works? Where do you think human value lies in such creations?
**Q22:** How do you feel about the statement “AIGC is changing the rules of the industry”? Has AIGC challenged or reshaped your identity as a creator?

### Part V: Conclusion and Outlook

**Q23:** Overall, what is your outlook on the future development of AIGC technology at Xuanjia Technology and in the wider video content creation industry?
**Q24:** In your opinion, what more can companies do to better promote the application of AIGC technology in the future?
**Q25:** From your perspective, what core abilities should creative professionals strengthen in the AIGC era to adapt to changes?

**Closing Remarks:**
Thank you very much for taking the time to share your experiences and insights. Your feedback is of great importance to our understanding of AIGC applications in the creative industries. If you have any further thoughts or additional input after the interview, please feel free to reach out to us. Thank you again!

# Interview Transcript

## Participant 1 (AI algorithm engineer at a technology company)

### Part I: Background & AIGC Usage Overview

**Q1.** Hello. I work as an AIGC algorithm engineer at **[a technology company]**. I have been in this role for **[several years]**. My position bridges technical and creative teams. Main duties include: contributing to the design and optimization of our **[in-house AIGC platform]**; integrating state-of-the-art AI into the production pipeline; offering AIGC solutions for creative projects; and training internal teams.

**Q2.** My daily toolkit is hybrid. The **[in-house AIGC platform]** is core for generating video from text or images. I also use **[third-party image generation tools]** for early visual exploration. During screenwriting, we use **[a large language model]** to support brainstorming. In addition, **[a rapid video editing tool]** helps with quick montage and automation for short-form content.

**Q3.** I use AIGC tools every day. They have permeated every stage of my work, from ideation to execution. AIGC isn’t a one-off assistant; it’s part of the infrastructure of my job.

### Part II: Perceived Usefulness & Ease of Use (PU & PEOU)

**Q4.** The biggest value of AIGC—especially the **[in-house platform]**—is compressing time and cost from idea to image. It tackles core pain points: historically high asset-creation costs and long timelines (what once took weeks for complex shots can now yield drafts within hours); communication barriers (rapidly turning abstract text into moving visuals); and creative stimulation (producing unconventional visuals that catalyze ideas).

**Q5.** It boosts both efficiency and quality. For example, on a **futuristic-city** promo piece, AIGC produced hundreds of diverse concept frames and motion previews **[in a single working day]**. This cut the concept phase by **[a substantial proportion]** and increased richness and detail beyond expectations. It acted not only as an efficiency tool but also as a quality amplifier.

**Q6.** Two areas remain weak: (1) character motion and facial continuity in long shots—maintaining identity and emotion consistently is hard; (2) fine-grained physical interactions (e.g., fluid behavior, cloth tearing) still lack realism and often require traditional CG.

**Q7.** The **[in-house foundation model]** is vertically optimized. Fine-tuned on **[legally obtained, domain-specific audiovisual data]**, it yields more stable, controllable outputs in particular aesthetics (e.g., certain regional or genre styles) and can follow cinematic language. We are developing **character/scene locking** to ensure asset consistency across sequential content—crucial for professional pipelines.

**Q8.** The interface is creator-friendly and easy to start with. Mastery still has a learning curve, especially “prompt engineering”—learning to direct the AI precisely is an art requiring practice.

**Q9.** The biggest challenge is output unpredictability. I address this by: iterative prompt refinement; using image-to-image or video-to-video when precise control is needed (starting with roughs from traditional tools); and treating AI outputs as semifinished assets to be refined in post.

**Q10.** Training/support are solid, with regular sharing sessions and an internal knowledge base. I’d like more end-to-end, scenario-based workshops so people learn within full project flows rather than in isolated tips.

### Part III: Perceived Creative Enhancement (CEP)

**Q11.** AIGC greatly amplifies my creative process. It frees imagination from production cost constraints, enabling rapid validation of ideas once out of reach. By generating unexpected combinations, it often breaks cognitive ruts. It also lets me try formerly expensive style fusions—e.g., blending classical painting aesthetics with science-fiction—opening vast possibilities.

**Q12.** For story ideation and plotting, **a large language model** is a powerful assistant. We input a basic scenario and obtain multiple trajectories, conflicts, or stylized dialogue. It doesn’t “write for us,” but it broadens the writer’s option space.

**Q13.** Diversity is excellent; originality and personal style depend on the creator. The AI itself has no inherent style—its style emerges from training data and direction. With distinct prompt strategies, aesthetic curation, and post-processing, you can achieve a strong personal voice. AI is a brush; style comes from the person holding it.

**Q14.** Absolutely. It reduces repetitive labor substantially. For example, creating **animatics** used to require many sketches and assembly; now we can input a script into the **[in-house platform]** and quickly generate a draft with basic camera movement. This shifts most effort to top-level creative decisions (story rhythm, shot logic).

**Q15.** AIGC raises the creative floor and expands the exploration ceiling. It enables high-quality visuals even on tight budgets and offers an unprecedented lab for experimentation. It doesn’t equal higher art by itself, but it multiplies the paths to reach it.

### Part IV: Creative Autonomy Retention (CAR)

**Q16.** My control has shifted. Previously, I worked like a sculptor at the micro level; now I operate more like a director, setting rules and direction for the AI. I’ve traded pixel-level control for stronger control over style and iteration, which feels liberating.

**Q17.** I’m aware of the “black-box” nature but not overly concerned. It’s a capability trade-off: we surrender full process transparency for exploratory power. My job is to probe the box through testing, map its boundaries, and use post-production to steer outputs.

**Q18.** I don’t fear replacement, but roles will evolve. Highly routine tasks are at risk. Core creator value—taste, narrative sense, artistic judgment—becomes more important. Designers shift from software operators to AI conductors and creative strategists.

**Q19.** The future relationship is like navigator and engine. Humans set destination and emotional core; AI provides power and route options. AI autonomy will grow within the process, but final decision-making and meaning-making stay with human creators.

**Q20.** When outputs miss the mark, I treat them as serendipity—sometimes they spark better ideas. If unusable, I adjust prompts, switch models, or change random seeds. Tuning the AI is itself creative, guided by my aesthetics and narrative goals.

**Q21.** Authorship and credit should belong to human creators. AI is a tool (like a camera or brush). Human value spans the chain—from concept and prompt design to selection and integration into a coherent, emotive work. Humans imbue the result with soul and meaning.

**Q22.** The idea that AIGC is changing industry rules excites me. My creative identity isn’t threatened; it’s broadened. I’m not just an executor but a fusion of creativity and technology—an artist expressing through a new paradigm.

### Part V: Summary & Outlook

**Q23.** I’m very optimistic. AIGC will become standard in video creation, lowering barriers and fostering a flourishing content ecosystem. Within **[the organization]**, it will continue to permeate all stages, forming a highly intelligent, closed-loop production system.

**Q24.** Future improvements: (1) tighter toolchain integration for seamless data/flow; (2) a more systematic, intelligent enterprise knowledge base for rapid reuse of successes; (3) a culture of cross-boundary innovation, including internal creative challenges to spur disruptive ideas.

**Q25.** Core skills for creators in the AIGC era: (1) top-tier taste and aesthetic judgment; (2) effective communication with AI—excellent prompting and logical structuring; (3) powerful integrative storytelling—weaving AI-generated elements into moving narratives.

## Participant 2 (Founder/senior leader of ****the**** technology company)

### Part I: Background & AIGC Usage Overview

**Q1.** I am the founder and **[senior executive]** of **[the organization]**. My core responsibilities are setting strategy, aligning resources, building high-performing teams, and keeping us at the forefront of innovation. The organization was born from an anticipation of transformation in content creation.

**Q2.** I don’t operate AIGC tools daily like engineers or designers, but I engage at a strategic level: deeply involved in roadmap reviews for the **[in-house AIGC platform]**; regularly reviewing key demos and deliverables produced with **[the in-house platform and third-party tools]** to evaluate maturity and commercial potential; and personally test-driving breakthrough features to keep a direct feel for the tech.

**Q3.** My attention to AIGC is continuous. At the decision level, AIGC is central to our strategy discussions. Every major project must first answer: what role can AIGC play, and what disruptive value can it bring?

### Part II: Perceived Usefulness & Ease of Use (PU & PEOU)

**Q4.** AIGC—especially our **[in-house platform]**—reshapes the business logic of content production. It addresses the long-standing “impossible triangle” of quality, cost, and speed. The platform enables quality at or above traditional standards while compressing costs and timelines by orders of magnitude. It solves systemic, end-to-end issues, not just a single step.

**Q5.** Efficiency and quality gains are dramatic. Previously, to bid for a large project, we might dedicate a specialist team for weeks to produce a short concept piece—costly and risky. Now, using the **[in-house platform]**, a core creative team can generate multiple high-quality motion proposals aligned to a client’s initial idea **[within a very short timeframe]**. This is not just efficiency—it’s a business-model upgrade: we offer creative visualization and rapid validation, improving win rates and client satisfaction.

**Q6.** From a strategic view, current limitations are **[key challenges to be tackled]** rather than minor defects: e.g., controllable long-duration character consistency and complex story logic generation. These are focus areas for future R&D; solving them will deepen our technical moat.

**Q7.** The **[in-house model]** has advantages in three areas: (1) **unique data assets**—trained on **[legally cleared, carefully annotated, domain-specific audiovisual data]** for strong cinematic understanding and stylistic control; (2) **workflow integration**—designed to plug directly into professional post/3D/compositing tools for industrial-grade production; (3) **safety and governance**—enterprise-grade content review and IP-risk controls, which general-purpose models usually lack.

**Q8.** Our design philosophy is to let creators return to creation. We invest heavily in usability so directors and writers without technical backgrounds can onboard easily. Mastery still requires learning, but it’s essentially a new expressive language, not mere software complexity.

**Q9.** The biggest barrier isn’t technology but organizational inertia and workflow redesign. We address this through a top-down culture shift, consistent internal advocacy of AIGC’s strategic value, and dedicated incentives that reward teams for bold integration and breakthroughs.

**Q10.** Training is a strategic investment. Beyond regular tech training, we established an **AIGC Center of Excellence (CoE)**—a cross-functional virtual team of top talent tasked with exploring best practices, standardizing workflows, and scaling training. We plan to open parts of this capability to the wider ecosystem.

### Part III: Perceived Creative Enhancement (CEP)

**Q11.** AIGC has a catalytic impact on our creative process—more than inspiration, it releases immense energy by catalyzing our people’s imagination.

**Q12.** For story development, AIGC moves us toward data-assisted decision-making. Writers can use **[a large language model]** to simulate many narrative paths and relationships. This doesn’t replace writers—it broadens their field of view and provides evidence-informed references.

**Q13.** Originality depends on the user. AIGC is a powerful style engine and amplifier. We encourage seeing it as a customizable partner; through bespoke directives, aesthetic selection, and secondary creation, one can develop distinctive styles carrying individual or organizational signatures.

**Q14.** The core organizational benefit is freeing creativity from repetitive, low-value work. Previously, concept artists might spend most of their time executing; now, with AIGC, the ratio flips toward high-level ideation, world-building, and style definition.

**Q15.** AIGC lifts our creative baseline and powers our push toward higher artistic standards. Projects can start at visual levels once reserved for top-tier productions, letting us channel energy into story core and artistic expression.

### Part IV: Creative Autonomy Retention (CAR)

**Q16.** Creative control has not diminished—it has expanded. Creators are liberated from pixel-level manipulation and can now define rules, styles, and world evolution—higher-dimensional creative freedom.

**Q17.** I’m not worried about black-box aspects. Revolutionary tools are opaque early on. Our job is to build a rigorous control system—test, feedback, iterate—and “tame” the tool. I have confidence in our team.

**Q18.** Roles will evolve rather than be replaced. Routine roles will phase out, while high-value roles emerge (e.g., AIGC director, AI trainer, creative engineer). Our responsibility is to lead employees through this evolution so their skills appreciate in value.

**Q19.** The ideal collaboration is human-AI symbiosis, with creativity in the lead. AI is a powerful copilot and executor; humans steer, decide destinations, and imbue meaning. AI autonomy may grow, but it serves human-set objectives and values.

**Q20.** When outputs miss expectations, that’s the moment for human creativity to intervene. Each adjustment is a high-level conversation with the AI—a re-creation grounded in human aesthetics, experience, and emotion.

**Q21.** Our stance on attribution is clear: AIGC is a tool; copyright and credit belong to the human creators and their institutions. Human value lies in final decisions, aesthetic judgment, and the infusion of thought and experience.

**Q22.** AIGC changing the rules is the most exciting call of our time. Our goal is not to follow rules but to help define new ones. Creative identities are becoming stronger—we are evolving from content makers to creators in a new era.

### Part V: Summary & Outlook

**Q23.** I have a very positive outlook on AIGC in video content creation. It is a deep industrial shift that will empower individuals, reshape enterprise production, and spawn new content forms.

**Q24.** Our future focus: (1) sustained investment in core tech (e.g., the **[in-house platform]**) to deepen technical defensibility; (2) building an open AIGC ecosystem with partners, academia, and independent creators; (3) leading standards and ethics to ensure beneficial, sustainable development.

**Q25.** Key capabilities for creators: (1) strong aesthetic decision-making to choose well among many AI-enabled options; (2) cross-domain integration across technology, art, and business; (3) inexhaustible curiosity and courage to embrace change.

Below is an **anonymized English translation** (continued). All potentially identifying details (people, exact ages, addresses, dates, institutions, company/product names, etc.) have been removed or generalized.

## Participant 3 (Video editor at ****the**** technology company)

### Part I: Background & AIGC Usage Overview

**Q1.** Hello, I am a video editor at **[the organization]** with **[multiple years]** of experience. My main responsibility is post-production editing for various video projects—selecting, ordering, and combining footage; shaping narrative through shot language, pacing, and sound design. In short, I turn scattered images into a compelling story.

**Q2.** AIGC tools are now regulars on my editing desk. Besides traditional NLEs **[general editing software]**, I use our **[in-house AIGC platform]** and **[an internal rapid-editing tool]**. The **[in-house platform]** helps me synthesize transitions, VFX shots, or establishing shots that would be costly or difficult to capture. The rapid-editing tool accelerates rough cuts for short social videos by auto-matching music and making preliminary edits. I also use AI audio utilities for speech enhancement and auto-captioning.

**Q3.** I’m using AIGC more and more—now in almost every project. I reach for it whenever footage is missing, I need a creative transition, or I want to scaffold an edit quickly. The **[in-house platform]** has become an indispensable source of high-quality semi-finished assets.

### Part II: Perceived Usefulness & Ease of Use (PU & PEOU)

**Q4.** AIGC greatly expands my asset library and creative methods, solving key pain points:

**Footage gaps:** If I need, say, a dusk time-lapse from a specific angle that wasn’t shot, I can generate it via the **[in-house platform]**, controlling light, weather, and style to match context.

**Monotone transitions:** Beyond cuts/dissolves/wipes, I can synthesize imaginative transitions (e.g., an eye morphing into a starfield), boosting visual impact.

**Efficiency:** The rapid-editing tool handles rough sync and selects, freeing my time for fine-cut narrative and emotion.

**Q5.** Efficiency and quality gains are obvious. For a product promo, the director wanted a seamless zoom from micro-level circuitry to a macro cityscape—traditionally very costly CG. Using the **[in-house platform]**, I specified camera moves and style references to generate a high-quality motion shot. With careful integration and grading, it realized the vision at a fraction of the usual time and cost and became the highlight of the piece.

**Q6.** Where AIGC is less helpful: understanding human emotion. It can cut to rhythm, but it struggles with micro-expressions and meaningful pauses. For fine emotional pacing, I rely on professional judgment—AI can cut “correctly,” but rarely “beautifully.”

**Q7.** From a user perspective, the **[in-house model]** stands out for cinematic quality and customizability. Many generic outputs look flashy but feel like VFX piles. Here, light, texture, and camera motion align better with film standards, and I can fine-tune details (e.g., bokeh softness, a particular auteur-style grade), which is critical for professional editors.

**Q8.** Interfaces are intuitive with low entry barriers: input idea → adjust parameters → generate. For people used to creative software, onboarding is fast. Design is trending toward invisibility so we can focus on creativity.

**Q9.** Main challenge: randomness. If I want motion from A to B, the AI might go A to C. I mitigate by iterating prompts, sampling multiple versions, and then splicing usable segments with editorial craft. I treat it as a source of high-quality semifinished material, not a one-click button.

**Q10.** Training/support are solid—regular showcases and a responsive internal community. I’d love more advanced workshops for editors on seamless compositing and grading of AI-generated with live-action footage.

### Part III: Perceived Creative Enhancement (CEP)

**Q11.** AIGC has liberated my imagination. I used to “find stories” within limited footage; now I can “create stories” in a space of near-infinite possibilities. I think about what I need, then try generating it—shifting from passive combination to active creation.

**Q12.** Though I’m in post, AIGC opens narrative options—e.g., generating flashbacks or hallucinations to deepen interiority without reshoots or expensive CG.

**Q13.** AIGC itself isn’t original; it’s like an erudite but opinion-free scholar. Originality emerges through editorial authorship—curation and montage that align outputs to my narrative intent and personal style.

**Q14.** For trailers/shorts, AI preselects highlights via tags (emotion, aesthetics, motion), saving at least half my initial sift time and letting me invest energy in suspense and payoff design.

**Q15.** It’s a catalyst and amplifier of creative quality: more and better “pigments” for a richer, layered “painting,” breaking physical constraints so visuals better serve story.

### Part IV: Creative Autonomy Retention (CAR)

**Q16.** My control is more concentrated on the core. I relinquish some low-level asset hunting but gain stronger command of pacing and emotion. Less a “material organizer,” more an “architect of story,” with better bricks at hand.

**Q17.** I don’t fear the black box. Editors constantly reconcile uncertainties (director intent, performances). AI is a new, temperamental collaborator to be understood and steered.

**Q18.** Routine, mechanistic tasks will be automated, highlighting the editor’s core value: narrative sense, rhythm, and taste. I aim to evolve from software operator to AI-literate narrative strategist.

**Q19.** Think of AI as a gifted assistant editor needing direction. It can handle rough cuts, organization, and patterned tasks; I, as lead editor, own artistic direction, emotional arcs, and final decisions.

**Q20.** If outputs miss the mark, I either regenerate or mine “errors” for happy accidents. Choosing if/where/how to use a shot is authorship.

**Q21.** Attribution belongs to the editor/team. AI outputs are raw material; meaning arises from human selection, arrangement, and integration.

**Q22.** Changing rules feels liberating—fewer resource-bound constraints, more editorial freedom. My identity is strengthened because storytelling skill matters even more.

### Part V: Summary & Outlook

**Q23.** I’m bullish: AIGC will become as ubiquitous as office tools, a foundational layer like non-linear editing—raising both efficiency and imagination.

**Q24.** We could build a smarter, shareable internal AIGC asset library—tagged, reusable transitions/effects—to avoid reinventing the wheel.

**Q25.** Editors should strengthen: (1) narrative construction; (2) open, experimental learning; (3) aesthetic integration—harmonizing diverse AI and live-action materials within a coherent style.

## Participant 4 (Professor of film/TV at ****[an arts university]****)

### Part I: Background & AIGC Usage Overview

**Q1.** I’m a professor with **[decades-long]** experience in film theory/practice, focusing on digital media and narratology, and on how technological shifts impact film language and industry. I teach directing, digital post, and an advanced course on computational art and generative moving images—grounding students in theory while cultivating critical thinking and innovative practice.

**Q2.** In research/teaching, I use widely available AIGC tools: guiding students to employ **[public image generators]** for concept/world-building; **[text/image-to-video tools]** for rapid animatics and experimental shorts; and **[a large language model]** in screenwriting courses for outlining, structural exploration, and dialogue polishing.

**Q3.** My use is research- and teaching-driven—frequent and systematic. I test platforms weekly to map potentials/limits and feed findings into lectures and papers. Students are required to use these tools at specific stages, making AIGC a routine, important part of the curriculum.

### Part II: Perceived Usefulness & Ease of Use (PU & PEOU)

**Q4.** AIGC dramatically lowers the threshold for “visual thought experiments,” addressing constraints of resources and technical barriers in film education.

**Q5.** It boosts pedagogy and ignites creativity. For directing classes, students now submit AI-assisted visual style decks and concept teasers, making expression more concrete and critiques more precise.

**Q6.** Risks: students may neglect craft. One-click spectacle can erode motivation to master lighting, composition, camera movement, teamwork, and on-set problem solving.

**Q7.** Public models excel in accessibility and breadth but lack deep film literacy—blocking/staging rules, axis of action, montage logic. Outputs can be fragment piles rather than shot language serving narrative. Education must fill that gap.

**Q8.** Ease of use is double-edged. Typing prompts is trivial; achieving precise control demands aesthetic literacy, visual culture, and exact language.

**Q9.** Biggest challenge: handling unpredictability and stylistic sameness. I reframe AIGC as a catalyst, not a final product generator. Students must incorporate human editorial/compositing/grading—or even live-action overlays—to inject authorship and break plastic uniformity.

**Q10.** Institutions need systemic reform: curriculum-wide AIGC integration, compute resources, and a cross-disciplinary AI+Art research center with clear ethics.

### Part III: Perceived Creative Enhancement (CEP)

**Q11.** AIGC acts as a visual association engine, offering many divergent options per concept and helping break cognitive ruts.

**Q12.** In writing, **[a large language model]** serves as tireless doctor/sparring partner—questioning logic from different characters’ POVs or continuing in specific styles. It’s for debate and refinement, not ghostwriting.

**Q13.** AI isn’t original; originality lies in how creators use it—distinct prompts, unique curation, montage-driven recombination.

**Q14.** It frees time from asset grind to story, rhythm, and core ideas—allowing teaching to emphasize directing and narrative intelligence over software.

**Q15.** AIGC raises technical floor, not artistic ceiling. Art rests on humanistic depth and emotional power.

### Part IV: Creative Autonomy Retention (CAR)

**Q16.** Control shifts from micro to macro—from physical minutiae to rule-setting, style definition, and selection. A move from craftsperson to architect.

**Q17.** The black box is a valuable epistemic topic—students should study bias and logic, building media literacy and the ability to collaborate with partially controllable systems.

**Q18.** Replacement anxiety is real for repetitive roles, but core capacities—taste, empathy, complex narrative construction, cross-cultural collaboration—remain irreplaceable.

**Q19.** Preferred model: the Centaur—human strategy/ethics/decisions + AI tactics/execution.

**Q20.** Misfires are teachable moments: diagnose prompt precision vs. model drift; explore whether “errors” open better directions. Students remain authors by judging, selecting, and meaning-making.

**Q21.** Authorship stems from human originality in thought and expression. Humans conceive, select, and construct meaning; AI remains a tool/medium, not an independent author.

**Q22.** The changing landscape heightens my sense of mission: from transmitting established knowledge to co-exploring unknowns with students.

### Part V: Summary & Outlook

**Q23.** Cautious optimism: democratization alongside risks—homogenization, echo chambers, ethics. The future is human-AI symbiosis.

**Q24.** Universities should: break silos; update curricula dynamically; and elevate media ethics and critical thinking to core requirements.

**Q25.** Core capabilities for students: (1) narrative and humanistic literacy; (2) problem-framing and questioning; (3) integration/curation—combining AI, live-action, and archives into coherent, meaningful wholes.

## Participant 5 (Animation lecturer at ****[an arts institute]****)

### Part I: Background & AIGC Usage Overview

**Q1.** I’m a lecturer with **[over a decade]** of teaching experience, transitioning from hand-drawn animation to 3D and digital media. I teach motion principles, character design, storyboards, and capstone supervision—helping students master animation as a time-based art and explore expression across media.

**Q2.** AIGC is now unavoidable in teaching and personal research. We guide students to use **[public image generators]** for character/scene/style exploration; **[text/image-to-video tools]** for key dynamic shots in boards and previews; and **[workflow variants based on control or motion-guidance modules]** to apply generated styles to existing sequences.

**Q3.** I engage with AIGC weekly. It’s embedded in course design and assignments—not for commercial deadlines but as a pedagogical lab to probe boundaries, pros/cons, and how it reshapes our understanding of animation.

### Part II: Perceived Usefulness & Ease of Use (PU & PEOU)

**Q4.** AIGC breaks barriers between productivity and imagination, addressing:

**High art-skill threshold:** Students with strong ideas but weaker draftsmanship can present concepts with higher visual quality.

**Long cycles:** It compresses time for early art and some mid-stage assets.

**Style exploration limits:** Students can test many styles in a day.

**Q5.** Results are significant. In character design, students now deliver mood boards, world-based variants, and dynamic poses as a coherent visual report using AI-assisted processes—deepening breadth and depth.

**Q6.** A major caveat: AI doesn’t understand the **12 Principles of Animation**. Motion often lacks weight, elasticity, and timing; characters move like paper cutouts. Over-reliance risks losing the core craft—breathing life into performance.

**Q7.** Public models excel at style transfer and still-image generation—great concept artists—but lack temporal logic. Animation is about inter-frame relations and performance; current models trained largely on stills don’t grasp anticipation, follow-through, or arcs.

**Q8.** Interfaces seem “zero-barrier,” but true mastery is hard. The challenge isn’t button-pushing; it’s aesthetic judgment and art direction—choosing from floods of outputs and steering prompts toward intent.

**Q9.** To prevent students from being subsumed by default AI aesthetics, I enforce a **hybrid workflow**: AI outputs are inputs, not final products. Students must import them into professional tools for repainting, layering, and re-creation, embedding their own design thinking and mark-making.

**Q10.** We’re catching up institutionally with electives and workshops, but need systemic integration across the program and better compute resources.

### Part III: Perceived Creative Enhancement (CEP)

**Q11.** AIGC is an inexhaustible visual muse, helping students leap beyond comfort zones with unexpected combinations—valuable in early brainstorming.

**Q12.** It rapidly converts scripts to visual drafts so students can judge continuity and pacing more concretely.

**Q13.** AI recombines data; originality emerges in how animators select and transform it. The clay is AI’s; the sculptor is the student.

**Q14.** Productivity gains are clear for backgrounds: AI can generate high-quality plates quickly, with students focusing time on performance animation—the essence of “animate.”

**Q15.** It boosts production value but not art level by itself. A dazzling backdrop can’t save stiff animation or weak stories.

### Part IV: Creative Autonomy Retention (CAR)

**Q16.** Control shifts from micro-execution to macro-direction—setting rules, selecting results, and integrating layers.

**Q17.** Animators are used to finding freedom within constraints; AI unpredictability is just a new, interesting constraint to master.

**Q18.** “Draftsperson” roles may fade; performance designers and storytellers won’t. Core values—performance design, timing/rhythm, dynamic storytelling—are AI’s weak spots and our educational core.

**Q19.** Ideal collaboration: lead animator + AI assistant. Humans design keyframes, emotions, and core dynamics; AI supports in-betweens and rapid look development.

**Q20.** Misaligned outputs become creative starting points—diagnose, modify, and re-create. Final inclusion and placement are human authorship.

**Q21.** In animation, authorship follows performance design. Without human timing and expression, AI-generated characters are lifeless puppets; the animator who gives life is the author.

**Q22.** Changing rules bring excitement and urgency. My role expands from transmitting craft to guiding exploration—demanding continual learning and reflection on animation’s essence.

### Part V: Summary & Outlook

**Q23.** AIGC will transform animation—enabling more personal, stylized indie works and reshaping studio pipelines for efficiency and flexibility.

**Q24.** Education should pivot from “teaching software” to “teaching workflow and creative thinking,” fostering cross-disciplinary collaboration with computing, theatre, literature, etc.

**Q25.** Students should strengthen:

**Foundational principles** (the 12 rules underpin judgment and AI guidance);

**Art direction and aesthetic curation** (unifying diverse materials into a coherent world);

**Narrative and performance design** (story and character are the enduring human core).

Below is an **anonymized English translation** (continued). All potentially identifying details (people, exact ages, addresses, dates, institutions, company/product names, etc.) have been removed or generalized.

## Participant 6 (Lecturer in digital media arts at ****[an arts institute]****)

### Part I: Background & AIGC Usage Overview

**Q1.** Hello. I am a lecturer in the School of Animation/Media at **[an arts institute]**, teaching and researching digital media arts. My academic background sits at the art–technology intersection; my doctoral work focused on 3D animation and digital media. Digital media arts is inherently exploratory: we treat digital technology itself as artistic medium and language. My role is to guide students to understand how code, algorithms, and data become brushes and pigments for expressing ideas and crafting experiences.

**Q2.** In teaching and personal practice, AIGC tools are core media for creation and research. Our toolkit goes far beyond text-to-image. In addition to **[public image generators]**—often customized via APIs or control modules—we make extensive use of **[creative coding platforms]** with AI models called through APIs as generative nodes; **[video-generation tools]** that interoperate with other software for real-time style transfer or pose recognition; **[AI audio tools]** for generative music and audiovisual soundscapes; and **[large language models]** not only for copy but also to script dialogue logic, poetry, and interactive behaviors within installations.

**Q3.** My engagement is immersive: I read papers daily, test new models/workflows, and rapidly convert them into case studies. At least half of my assignments require students to integrate AIGC in some form, so it is part of our daily academic life.

### Part II: Perceived Usefulness & Ease of Use (PU & PEOU)

**Q4.** AIGC solves a foundational issue: it democratizes generative art beyond a small group of programmers. Previously, complex emergent works demanded deep coding skills. AIGC lowers the barrier so students can focus on why to generate and how to interact with outputs, not only how to code them.

**Q5.** In my interactive-art course, one capstone involved an installation where visitors spoke a recent dream into a microphone; speech recognition converted it to text; a language model distilled poetic keywords; those drove a generator to produce flowing, abstract visuals projected onto scrim. Here, AIGC served as the core translator/processor connecting audience, dream, and image—hard to imagine pre-AIGC.

**Q6.** Traps include homogenized aesthetics and “predictable unpredictability.” Models tend to reproduce popular styles in training data, pulling student work toward glossy sameness. Randomness brings serendipity but rarely produces truly subversive, deep expression unaided. It excels at synthesis, not invention.

**Q7.** The strength of general models is broad knowledge and generalization. For digital media artists, their value lies in plasticity and integrability. We seldom use web UIs; we care about APIs—treating the model as an external brain/visual engine embedded in our interactive systems or installations. The point is not what the model itself can do but what systems we build with it.

**Q8.** Usability shows **low floor, high ceiling**. Anyone can generate an image; creating a truly valuable DMA artwork demands designing prompt sequences, chaining models, writing code to control/process outputs, and crafting thought-provoking interaction—an interdisciplinary climb.

**Q9.** The biggest teaching challenge is moving students from playing with AI to thinking/creating with AI. I emphasize process and concept: deliverables must include a creation log, model-tuning process, failed attempts, and—crucially—an articulation of why AI is used for the theme. I encourage “misuse/abuse” of AI—finding artistic possibilities in errors and glitches.

**Q10.** Institutions should build cross-disciplinary AI-art labs that unite art, computing, and humanities in one space, plus provide cloud GPU resources to enable deeper, larger-scale experiments.

### Part III: Perceived Creative Enhancement (CEP)

**Q11.** AIGC elevates the creative dimension: from mere inspiration to a nonhuman collaborator we can converse with, co-evolving the work. We shift from designing objects to designing systems that grow.

**Q12.** Narratives are often non-linear. AIGC helps build possibility spaces—e.g., in VR, the system can generate environment changes and branches in real time based on gaze and behavior. AI becomes a dynamic world-builder.

**Q13.** In DMA, originality resides in the designed system and rules. If an artist designs a unique mechanism that interacts with audiences and calls AI to generate, the system is the original work; images are snapshots. Personal style is the “unique manual” you design for using AI.

**Q14.** AIGC lets us focus on higher-level questions. Instead of drilling shader/particle code for spectacle, we explore cultural metaphors and the meanings of human–AI interaction.

**Q15.** It greatly enriches media language and expressive layers. Artistic level depends on conceptual depth and apt media use; AIGC provides a powerful new language that can raise conceptual expression when used well.

### Part IV: Creative Autonomy Retention (CAR)

**Q16.** Control shifts from pixel-level absolutism to guiding probabilities and processes—like moving from painter to gardener. You can’t control each leaf, but you nurture the ecosystem. It’s more generative and challenging—central to DMA practice.

**Q17.** The black box invites inquiry. The box itself becomes an object for exploration and critique; many projects revolve around dialogue with it—probing bias, dreams, and presenting that exploration as art.

**Q18.** Replacement anxiety is milder in DMA because we don’t train “button-pushers.” Those without ideas may be displaced; critical, system-designing artists gain opportunity.

**Q19.** Ideal collaboration is a symbiotic creative collective: humans provide intent, ethics, and aesthetic judgment; AI offers computation, variation, and nonhuman viewpoints.

**Q20.** Misaligned outputs mark the moment art happens—through selection, rejection, and transformation, artists inject new information that steers subsequent generation. The feedback loop is creation.

**Q21.** Authorship is challenged here; many works decenter it. If defined, authorship belongs to the person who designs the generative system and interaction rules. Humans build the conceptual container; AI fills it.

**Q22.** The “rule-changing” shift means the future long discussed in class has arrived. Our field moves to the forefront of art–technology fusion, reinforcing my professional identity.

### Part V: Summary & Outlook

**Q23.** AIGC will be ubiquitous yet invisible—like electricity—underpinning digital media. The distinction “AI art” will fade as most digital art becomes AI-aided.

**Q24.** We must cultivate AI literacy and algorithmic critique: how models are trained, embedded biases, decision logics, and societal impact.

**Q25.** Students should develop:

**Conceptual construction & philosophical inquiry** (what to express matters most);

**Cross-media system design & integration** (directing code, hardware, AI, space, sound into one experience);

**Critical technical understanding & ethics** (responsible, reflective innovation).

## Participant 7 (Photographer at ****[a media company]****)

### Part I: Background & AIGC Usage Overview

**Q1.** Please introduce your role, duties, and tenure.
I’m a photographer at **[a media company]**, with **[multiple years]** of experience. I handle visual realization across projects—from planning and on-set work to preliminary post organization—adapting to varied styles.

**Q2.** What AIGC tools/platforms do you use?
I use **[public image generators]** for concept exploration; **[video-generation tools]** for early concepting; **[AI upscaling/enhancement tools]** or neural engines for quality improvement; and **[large language models]** for copy and script polishing. We mix and match as needed.

**Q3.** Frequency/duration?
I use AIGC almost daily—1–2 hours for concepting in preproduction, and **[regular short sessions]** for post enhancements. It’s an indispensable assistant.

### Part II: Perceived Usefulness & Ease of Use (PU & PEOU)

**Q4.** Biggest benefits/pain points solved?
Rapid concept validation and inspiration; clearer communication of visual intent; quality lift on footage; overall efficiency gains and cost reduction.

**Q5.** Example of efficiency/quality gains?
For food advertising, I first generated multiple concept boards with **[public image generators]**, cutting on-site trial-and-error. A day’s shoot became a half-day—roughly **[significant percentage]** faster. We also combined AI-generated virtual backdrops with live action to reach aesthetics otherwise hard to achieve.

**Q6.** Less helpful areas?
Video coherence and fine control remain weak; outputs can feel templated or average; abstract concepts are hard to concretize faithfully.

**Q7.** On model advantages?
We mainly use general models: broad knowledge, multimodal inputs, active communities/fast iteration, and flexible composability—useful as a “creative brain” for references and ideas.

**Q8.** Usability/learning curve?
Interfaces vary: some easy, others parameter-heavy. Overall **low floor, high ceiling**—easy to start, hard to master for high artistry.

**Q9.** Difficulties/solutions?
Prompt precision and expectation mismatch; I iterate prompts, study exemplars, treat deviations as inspiration, and apply manual post. Compute cost is managed via planning; fragmented learning solved through communities and internal groups.

**Q10.** Training/support & improvements?
Support is decent with encouraged learning. Improvements: systematic training, internal knowledge base, more hardware/cloud resources, cross-team pilots.

### Part III: Perceived Creative Enhancement (CEP)

**Q11.** Impact on creativity?
Major inspiration boost—an “infinite visual dictionary” that unlocks new possibilities and composition/style experiments.

**Q12.** Role in story development?
I don’t write scripts, but AIGC helps teams visualize story concepts early; language models assist multi-angle plotting and dialogue polishing, improving overall efficiency.

**Q13.** Originality/diversity?
Diversity is excellent; originality is advanced recombination. Personal style emerges via prompt craft, selective curation/reconstruction, and injecting human intent.

**Q14.** Shift toward higher-value ideation?
Yes—e.g., generating location plates for commercials saves scouting and shooting time, letting me focus on deep creative discussions and challenging live-action shots. I move from executor to visual strategist.

**Q15.** Contribution to artistic level?
A catalyst/booster that expands creative boundaries and iteration speed; reaching higher artistry still requires deep human involvement and concept-driven use.

### Part IV: Creative Autonomy Retention (CAR)

**Q16.** Change in control?
Control shifts from micro to macro—directing, guiding, and making final choices—yielding greater freedom and higher-level authorship.

**Q17.** Black-box concern?
Initial concern faded. Understanding patterns and focusing on outcomes, I welcome unpredictability as a creative catalyst; human final review remains essential.

**Q18.** Impact on roles/skills?
Roles are reshaped and upgraded. Routine tasks may be automated; creators evolve into visual directors and AI collaborators. Tool-only operators risk displacement; critical, aesthetic, and innovative practitioners gain value.

**Q19.** Human–AI role split & future autonomy?
A symbiotic partnership: humans set intent and judge; AI executes and sparks ideas. Autonomy will grow within human frameworks; AI stays a tool, not an independent creator.

**Q20.** Handling mismatched outputs; creation vs. adjustment?
I refine prompts/parameters, salvage unexpected results, and integrate manually. This is co-creation, not mere adjustment—creativity lies in problem-posing, selection/reconstruction, and injecting human spirit.

**Q21.** Attribution and human value?
Attribution should primarily belong to human creators; AI is a tool. Human value lies in concept formation, system integration, aesthetic judgment, and ethical responsibility.

**Q22.** Changing industry rules & identity?
I’m excited—efficiency rises, barriers fall, forms diversify. My identity shifts from “shooter” to “visual world-builder” using camera language plus AI, centering creative leadership.

### Part V: Summary & Outlook

**Q23.** Outlook for **[the company]** and the industry?
Highly optimistic: deeper integration internally; broader democratization industry-wide; human–AI collaboration as norm; new roles emerging; ongoing copyright/ethics challenges to address.

**Q24.** How can organizations improve AIGC adoption?
Build systematic training; internal knowledge/case bases; invest in hardware/cloud; foster cross-team pilots; track frontier tech; strengthen ethics education.

**Q25.** Skills to develop in the AIGC era?

**Creative direction & concept design** (from executor to thinker; precise prompting).

**Cross-disciplinary system integration & iterative optimization** (blend AIGC with traditional tools; design efficient workflows; learn fast).

**Critical thinking & ethics** (discernment, quality evaluation, societal impact).

**Interview Closing.**
Thank you for your time. The AIGC wave is reshaping the field, and I’m excited to be part of it. I hope these reflections help your research.

## Participant 8 Head of the Department of Digital Visual Communication, [a university]

I am the head of the Visual Communication Design Department at Jinling University of Science and Technology. I hold a PhD and have been teaching for seven years. My duties include curriculum planning, course development, faculty management, and teaching core courses in visual communication. I also conduct research on AIGC in design and guide students’ graduation projects and innovation practice. In teaching, research, and personal creative work, AIGC tools are indispensable. I use Midjourney and Stable Diffusion for image generation, RunwayML and Pika Labs for video, Adobe Firefly for workflow efficiency, and ChatGPT or Claude for text. I engage with these tools almost daily, especially when preparing lessons, initiating research projects, or mentoring student work, which significantly boosts efficiency and creativity.

AIGC greatly benefits visual communication by rapidly generating ideas, speeding prototyping, lowering skill barriers, and broadening expressive possibilities. In my “Brand Identity Design” course, for instance, students quickly generate logo sketches and context visuals, shortening the design cycle. In research, AIGC provides diverse visual cases to strengthen analysis and allows students to exceed the limits of hand-drawing. However, it still struggles with precision, transparency, cultural nuance, and raises copyright and ethical concerns. Compared to traditional tools, AIGC offers essentially limitless ideation at near-zero cost, intuitive text-to-image/video pipelines, fast style adaptation, intelligent inpainting, and high-speed iteration that transform traditional design processes.

Ease of use varies. Midjourney is beginner-friendly, while Stable Diffusion is complex and parameter-heavy. This reflects a “low floor, high ceiling” learning curve: easy entry but difficult mastery. Students and I often face challenges with prompt accuracy, deviation from expectations, compute and time costs, and fragmented learning resources. We address these by refining prompts, reframing errors as inspiration, optimizing compute use, and building internal learning communities. The university supports experimentation with lectures and workshops but needs a systematic curriculum, internal knowledge base, stronger compute investment, closer industry partnerships, and emphasis on copyright and ethics education.

Creatively, AIGC is like an infinite visual dictionary that sparks unexpected ideas and overcomes technical or budget limits. It offers new angles for design ideation, variation, and style, acting as a “creative accelerator” and “visual explorer.” While diversity is strong, originality often lies in advanced recombination. Through unique prompting, selective curation, and the infusion of human intent and meaning, I and my students achieve personal style and distinctiveness. This allows us to focus more on meaningful creative thinking while reducing repetitive work. AIGC elevates the overall artistic quality of design output.

Working with AIGC shifts my control from micro execution to macro direction and final selection. Like a director or gardener, I shape growth through prompts and parameters. Early concerns about its black-box nature faded as I learned to adapt and refine results; sometimes unpredictability even enriches creativity. The technology reshapes rather than replaces the field. Routine tasks may diminish, but deep creative, strategic design roles will grow in value. For educators and students, cultivating critical thinking, aesthetic judgment, and innovative use of AI is crucial. I envision human–AI collaboration as a partnership where humans provide intent, judgment, and ethics, while AI delivers speed, inspiration, and variation. When outputs miss expectations, I repurpose or modify them, seeing this as creation, not adjustment. Authorship belongs to human creators, with AI as a tool; human value is reinforced in conceptual design, integration, aesthetic choice, and ethical reflection.

Looking ahead, I am optimistic about AIGC in visual communication and the broader industry. It will deeply embed in teaching and research, foster versatile talent, broaden content diversity, and create new roles around human–AI collaboration. Universities should implement coherent curricula, provide resources and compute, enhance faculty development, expand industry cooperation, and emphasize cross-disciplinary research and ethics. In the AIGC era, creative workers need three core abilities: strong conceptual and design leadership, cross-disciplinary integration and iterative optimization, and critical and ethical literacy. These skills will move us from executors to thinkers, ensuring technology serves human progress and creativity.

## Participant 9 (VFX Artist at a Media Company)

I am a visual effects artist at a media company in Nanjing and have worked in the field since 2017. My responsibilities span VFX design and production for commercials, promos, and digital content, including 3D modeling, look development and shading, particle and fluid simulations, and final compositing. My job is to turn the director’s and creative team’s ideas into striking images on screen.

In daily production, AIGC tools have become indispensable. I primarily use Midjourney and Stable Diffusion for rapid concept art and style exploration, which quickly supply extensive visual references. For 3D asset creation I experiment with AI aids such as 2D-to-3D conversion. AI also supports footage enhancement, denoising, and light-field simulation, and I rely on AI plugins for rigging and facial capture. I use these tools almost every day, especially for early look development and for late-stage detail polish, which significantly accelerates ideation and execution.

AIGC brings major benefits to VFX by enabling fast concept exploration, speeding prototype builds and complex scene layout, and widening the palette of artistic expression. On a fantasy project, for example, we can rapidly generate concept variations for creatures, magic effects, or otherworldly landscapes, sharply shortening the design cycle and avoiding costly redraws. It can also help produce background elements like foliage and rocks and even rough particle and fluid ideas, boosting previs and production efficiency.

Despite its strengths, AIGC is not yet reliable for film-final assets. AI-generated models may be overly dense with poor topology and require extensive manual cleanup; AI-driven fluids or particles often fall short of physically believable, feature-film standards. Copyright, data privacy, and style homogenization remain concerns. Compared with traditional DCC-only workflows, however, text-to-visual generation delivers a huge option space at low marginal cost and high speed, freeing VFX artists to focus on higher-value decisions.

Ease of use varies by tool. Concept generators like Midjourney are easy to start with simple prompts, while AI for 3D generation or advanced simulations still involves complex parameters and workflows that demand deeper learning. Our main challenges are precise control over fine details and temporal continuity, seamless integration of AI material with live-action footage, and keeping pace with constant model updates. We respond with tighter prompt engineering, finishing work inside standard DCC suites, and active internal knowledge sharing. The team would benefit from structured, hands-on AIGC-for-VFX training, more high-performance compute, and closer collaboration with AI vendors to tailor pipelines.

AIGC strongly amplifies creativity. It works like an endless visual library: with a few keywords and style cues it produces large sets of concepts and elements that escape habitual thinking and surface unexpected compositions, materials, and lighting. Ideas that were once limited by time, budget, or technology can now be validated quickly, expanding our expressive range.

For ambiguous briefs, AIGC provides fresh angles on design ideation, variation, and style testing. We can instantly generate distinct visual directions—say, a rain-soaked cyberpunk city or a derelict starship drifting through space—then evaluate possibilities and discover new entry points. Style exploration becomes an almost real-time preview, unlocking imagination rather than constraining it.

Working with AIGC shifts control from pixel-level handcraft to macro-level orchestration and precise guidance. I act more like a visual director, steering AI to produce initial materials through careful prompts and parameters, then applying professional judgment and traditional techniques to refine and integrate them. This gives me more freedom to invest in narrative intent, artistic expression, and complex technical challenges.

I am not worried about losing control over the final image. VFX excellence still depends on invention, problem-solving, and the art of seamlessly unifying disparate elements. AI supplies richer “parts” and faster “assembly,” but shaping them into something alive and affecting remains the VFX artist’s domain. The industry will favor those with strong aesthetics, technical integration skills, and innovative command of AIGC; rote executors may struggle, while artists who can harness AI to create spectacle will thrive.

I am highly optimistic about AIGC in VFX and across the screen industries. It will keep raising efficiency, lowering costs, and enabling more diverse and immersive visuals. For our studio, this means taking on more ambitious, high-concept projects at speed. Looking ahead, VFX artists should focus on three core capabilities: visual concept design that translates abstract ideas into AI-legible directions; integrated AIGC toolchains that mesh smoothly with DCC software; and rigorous taste and quality control to ensure AI outputs meet film-grade artistic and technical standards.

Thank you for the opportunity to share these experiences. I hope they offer useful perspective for your research. Our team will continue to explore the possibilities of AIGC in cinematic visual effects and bring audiences more compelling imagery.

## Participant 10 (Dean, University Animation School)

Background and AIGC Use
I serve as dean of a university animation school, overseeing curriculum reform, program development, faculty growth, research, and external partnerships. I closely track AIGC developments through reports, conferences, and internal feedback, and I personally test major tools such as Midjourney, Stable Diffusion, ChatGPT, and Claude. My goal is to judge how AIGC accelerates student creativity, improves research productivity, and fits our talent-training framework.

Perceived Usefulness
AIGC rapidly converts ideas into visuals, shortens concept, storyboard, and character-design cycles, and reduces repetitive labor. It broadens visual possibilities—surreal styles, textures, particles—and lowers skill barriers so more students can produce high-quality content. In class, students first generate varied concepts with AIGC, then refine them; in research, AIGC supplies diverse style samples; in production courses, it helps build complex scenes that surpass individual capacity.

Limitations
AIGC struggles with narrative continuity, precise keyframe control, consistent character forms, micro-expressions, and culturally nuanced meaning. Results can diverge from prompts, feel emotionally shallow, or show “AI look.” Legal, ethical, and data issues also constrain adoption.

Perceived Ease of Use
Entry is easy for friendly tools; advanced control in systems like Stable Diffusion demands knowledge of models, prompts, and pipelines. Effective practice requires prompt engineering, model tuning, post-processing, and tight integration with traditional DCC tools.

Creative Enhancement
AIGC acts as an idea accelerator and visual simulator, offering abundant compositions and style trials that make once-impractical approaches feasible. Its value grows when human creators curate, recompose, and infuse outputs with narrative intent, emotion, and cultural insight.

Creative Autonomy
Control shifts from pixel-level crafting to high-level direction. I guide prompts, models, and parameters, then finalize via editing and post. The “black box” is manageable through understanding, iteration, and human quality control; unpredictability can even spark new ideas.

Program and Industry Impact
AIGC will deeply embed across teaching, research, and production, raising efficiency and spawning new formats such as interactive or real-time animation. Routine tasks will be automated, while roles emphasizing concept, storytelling, aesthetics, and system integration gain value. We are building systematic curricula, shared resources and compute, industry collaborations, cross-disciplinary research, and robust ethics and IP education.

Key Competencies for Creators
Creators should strengthen high-level concept and narrative design, cross-tool workflow integration and rapid iteration, and critical and ethical judgment about data, bias, authorship, and social impact.

Closing
AIGC is becoming foundational infrastructure for animation education and practice. With strategic guidance and responsible use, it will expand creative possibilities while keeping human intention and judgment at the center.
