# Supplementary material for: The autonomy paradox in AI-generated content adoption: Creative-specific alternative to TAM model in China’s micro-short drama industry
Source: PLoS One. 2026 Jan 30;21(1):e0336166. doi: 10.1371/journal.pone.0336166 (PMC12858070; doi:10.1371/journal.pone.0336166)
Supplement: S7 File — Codebook for the CITAM dataset (N = 607, 21 variables), listing variable names, labels, coding, and notes. Includes demographics, IC, CAR (CAR4 reverse-coded), AI, and AUB items. Documents missing value treatment (none), data transformation, and scale composition, with all items measured on 5-point Likert scales. (DOCX) [file pone.0336166.s007.docx]

# CITAM Study Codebook

## Dataset Structure

- Sample size: N = 607
- Number of variables: 21
- File format: CSV

## Variable List

| **Variable Name** | **Variable Label** | **Value Coding** | **Notes** |
| --- | --- | --- | --- |
| ID | Participant ID | 1-607 | Unique identifier |
| Gender | Gender | 1=Male, 2=Female, 3=Other |  |
| Age | Age group | 1=Under 25, 2=25-34, 3=35-44, 4=45 and above |  |
| Experience | Creative experience | 1=Less than 1 year, 2=1-3 years, 3=4-6 years, 4=7+ years |  |
| Role | Primary role | 1=Scriptwriter, 2=Director, 3=Editor, 4=Independent creator, 5=Other |  |
| IC1 | AIGC revolutionary innovation | 1-5 scale | 1=Strongly disagree, 5=Strongly agree |
| IC2 | AIGC unique advantages | 1-5 scale | Same as above |
| IC3 | AIGC technology iteration | 1-5 scale | Same as above |
| IC4 | AIGC creative philosophy alignment | 1-5 scale | Same as above |
| CAR1 | Control core decisions | 1-5 scale | Same as above |
| CAR2 | Enhance expression ability | 1-5 scale | Same as above |
| CAR3 | Balance assistance with originality | 1-5 scale | Same as above |
| CAR4 | Limits creative autonomy | 1-5 scale | **Reverse-coded item** |
| CAR5 | Editorial/modification authority | 1-5 scale | Forward-coded |
| AI1 | Intention to continue using | 1-5 scale | Same as IC1 |
| AI2 | Recommend to peers | 1-5 scale | Same as above |
| AI3 | Learn advanced skills | 1-5 scale | Same as above |
| AI4 | Become important component | 1-5 scale | Same as above |
| AUB1 | Use in most projects | 1-5 scale | Same as above |
| AUB2 | Indispensable tool | 1-5 scale | Same as above |
| AUB3 | Actively explore functions | 1-5 scale | Same as above |

## Missing Value Treatment

No missing values (platform required complete submission)

## Data Transformations

CAR4 reverse coded: New value = 6 - Original value

## Scale Information

- **Innovation Compatibility (IC)**: Items IC1-IC4, measuring perceived alignment of AIGC with creative workflows
- **Creative Autonomy Retention (CAR)**: Items CAR1-CAR5, measuring perceived ability to maintain creative control (CAR4 reverse-coded)
- **Adoption Intention (AI)**: Items AI1-AI4, measuring behavioral intentions to adopt AIGC
- **Actual Usage Behavior (AUB)**: Items AUB1-AUB3, measuring actual AIGC usage patterns

All scale items use 5-point Likert scale (1=Strongly disagree, 5=Strongly agree)
