# Supplementary material for: The autonomy paradox in AI-generated content adoption: Creative-specific alternative to TAM model in China’s micro-short drama industry
Source: PLoS One. 2026 Jan 30;21(1):e0336166. doi: 10.1371/journal.pone.0336166 (PMC12858070; doi:10.1371/journal.pone.0336166)
Supplement: Attachment — Submitted filename: Response to Reviewers.pdf [file pone.0336166.s008.pdf]

Response to Reviewers

Manuscript ID: PONE-D-25-41907
Title: The Autonomy Paradox in AI-Generated Content Adoption: Extending the TAM Model in China's Micro-Short Drama Industry

Dear Editor Dr. Rafael Galvão de Almeida and Esteemed Reviewers,

We express our sincere gratitude for the opportunity to revise our manuscript and for the invaluable guidance provided throughout the review process. The constructive feedback has substantially strengthened our work and enhanced its contribution to the field.

We are particularly grateful to Editor Dr. Almeida for recommending the seminal works by Anantrasirichai and Bull (2022) and Hertzmann (2018). These references have been instrumental in better situating our research within the broader discourse on AI applications in creative industries. The Anantrasirichai and Bull framework helped us systematically categorize AIGC applications and articulate the quality concerns surrounding AI-generated content, while Hertzmann's philosophical perspective on computational creativity provided crucial theoretical grounding for our discussion of human agency in artistic creation. These additions have significantly enriched our theoretical foundation and practical implications.

We extend our heartfelt appreciation to both reviewers for their meticulous and scholarly evaluation of our work. **Reviewer #1: Dr. Mohammed Salah** provided exceptionally detailed methodological guidance that corrected several critical issues in our statistical reporting and theoretical positioning. **Reviewer #2** offered valuable insights into our theoretical framework, highlighting the need for deeper elaboration of complex theories. Their combined expertise has elevated the rigor and clarity of our manuscript substantially.

Point-by-Point Response to Revision Requirements

Editorial Requirements

1. Integration of Recommended Literature

> **Action Taken**: We have thoroughly integrated both recommended references throughout the manuscript. Anantrasirichai and Bull (2022) [29] is now cited in our new section "AIGC Quality Concerns and Creative Integrity" (lines 95-117) and extensively discussed in our quality control mechanism analysis (lines 697-723). Hertzmann (2018) [30] is cited in our theoretical framework

and discussion of human intentionality in creative processes (lines 105-109, 705-711, 850-853).

2. Quantitative Analysis of AI Quality Issues

Action Taken: We added a comprehensive section titled "AIGC Adoption as Quality Control Mechanism" (lines 697-723) that provides quantitative analysis of how the autonomy paradox serves as a natural barrier against AI-generated "slop." This section demonstrates how our CAR construct's negative moderation effects (β=-0.076; β=-0.078) may protect creative industry sustainability.

3. Creator Response to AI Quality Concerns

Action Taken: We expanded our discussion to address how creators cope with AI's impact on artistic meaning, particularly through our qualitative theme of "Creativity Amplification" and the strategic adoption patterns revealed in our findings.

Reviewer #1 Responses

1. Model Fit Indices Consistency

Issue: Discrepancy in TLI reporting between CFA and structural models

Action Taken: We have corrected and clarified all fit indices. The CFA model shows TLI = 0.985 (line 502), while the structural model shows TLI = 0.976 (line 525). The earlier report of 0.85 was a typographical error in our initial submission. All correct values are now consistently reported and supported by our statistical output in the appendices.

2. CAR Construct Definition and Measurement

Issue: Inconsistent naming and definition of the moderator construct

Action Taken: We have standardized the construct name as "Creative Autonomy Retention (CAR)" throughout the entire manuscript, including all tables, figures, and figure legends. The definition is consistently presented as "the perceived ability to maintain control and agency over creative processes" (line 162).

3. Effect Size Claims Correction

Issue: Inconsistency between f^2 values and variance explained claims

Action Taken: We have corrected our effect size reporting. The moderation effects show $f^2 = 0.006$ with corresponding $\Delta R^2 = 0.006$ (line 555), representing small but statistically significant effects that we now appropriately contextualize as "subtle but persistent" influences.

4. Sampling Description Alignment

Issue: Mismatch between planned and achieved sample distribution

Action Taken: We have clarified our sampling approach in lines 382-384, explicitly stating: "The achieved sample distribution differed from initial stratification plans due to varying response rates across professional roles, which may limit the generalizability of findings across all creator types in the micro-short drama industry."

5. Data Availability Compliance

Issue: Data sharing approach not meeting PLOS ONE requirements

Action Taken: We have deposited all de-identified survey data, codebook, and supporting materials in the Zenodo repository (https://doi.org/10.5281/zenodo.17216841) as detailed in our Data Availability Statement (lines 920-924).

6. Research Design Accuracy

Issue: Mislabeling of mixed-methods design

Action Taken: We have correctly identified our approach as an "exploratory sequential mixed methods design" throughout the manuscript (line 344).

7. Methodological Transparency Enhancements

Action Taken: We have added comprehensive details about variable centering procedures (lines 542-545), interaction term construction, and provided complete discriminant validity matrices and factor loadings in the appendices.

8.TAM Model Extension Issue

Issue: The manuscript claims to extend the TAM model, but it omits key constructs like PU and PEOU. The reviewer noted that this should be framed as an alternative model tailored to creative industries.

Action Taken: We have revised the manuscript to reflect that our model is a creative-specific alternative to the TAM model rather than an extension. We have updated the title to: "The Autonomy Paradox in AI-Generated Content

Adoption: Creative-Specific Alternative to TAM Model in China's Micro-Short Drama Industry" to clarify this distinction. The revised manuscript consistently refers to our approach as an alternative model throughout the text, aligning with the reviewer's suggestion.

Reviewer #2 Responses

1. Theoretical Framework Elaboration

Issue: Complex theories requiring more substantial explanation

Action Taken: We have significantly expanded our theoretical exposition, particularly for Self-Determination Theory (lines 223-240), providing detailed explanations of motivational regulation types and their relevance to creative contexts. We have also enhanced our discussion of cognitive fit theory, identity threat theory, and other complex concepts throughout the manuscript.

2. Theory Integration and Focus

Issue: Attempting to cover too many theories without sufficient depth

Action Taken: We have restructured our theoretical framework to provide deeper elaboration of core theories while maintaining appropriate scope. The expanded SDT section and enhanced integration with TAM and DOI frameworks now provide more substantial theoretical grounding.

Additional Improvements

Beyond addressing the specific requirements, we have made several additional enhancements:

Strengthened the connection between quantitative findings and qualitative insights through enhanced triangulation (lines 614-634)

Expanded practical implications for developers, managers, and policymakers (lines 757-799)

Enhanced our discussion of cultural contexts and future research directions (lines 811-829)

Improved clarity and consistency of statistical reporting throughout

Conclusion

This revision process has been invaluable in strengthening our manuscript. The integration of the recommended literature has enriched our theoretical foundation, while the methodological improvements have enhanced the rigor and transparency of our research. The expanded theoretical discussions have deepened our understanding of technology acceptance in creative industries.

We sincerely appreciate the insightful suggestions of Reviewer #1: Dr. Mohammed Salah and Reviewer #2, whose feedback has been instrumental in improving our manuscript. Their guidance has been invaluable in refining the quality and clarity of our work. We would also like to extend our heartfelt thanks to Editor Dr. Rafael Galvão de Almeida for his editorial support and for recommending key references that greatly enriched our theoretical framework. We believe the revised manuscript now offers a more refined and valuable contribution to the field.We hope the revised manuscript meets the high standards of PLOS ONE and look forward to your consideration for publication.

With sincere appreciation,
Chao Tang
Instructor
Department of Digital Media Arts
School of Animation
Jinling Institute of Technology
Nanjing, Jiangsu, China
Email: tangchao726@jit.edu.cn
